# Endemic Channel Parametrization in Dengue Surveillance: Methodological Assessment of Retrospective Windows, Outbreak Trends, and Zero-Case Periods in Colombia

**DOI:** 10.2196/79914

**Published:** 2026-05-08

**Authors:** Juan D Umaña, Juan Montenegro-Torres, Julian Otero, Maria Camila Tavera-Cifuentes, Natalia Niño-Machado, Catalina González-Uribe, Juan Manuel Cordovez, Mauricio Santos-Vega

**Affiliations:** 1 Grupo de Biología Matemática y Computacional (BIOMAC), Universidad de Los Andes, Carrera 1 # 18A - 12 Edificio A, Bogotá, Colombia, 57 6013394949 ext 2761; 2 Department of Community Medicine, Faculty of Health Sciences, UiT The Arctic University of Norway, Tromsø, Norway; 3 Centro de los Objetivos de Desarrollo Sostenible para América Latina y el Caribe (CODS), Universidad de Los Andes, Bogotá, Colombia; 4 Departamento Ciencias Biológicas, Universidad de Los Andes, Bogotá, Colombia

**Keywords:** endemic channel, epidemiological monitoring, dengue, disease surveillance, disease outbreaks, early warning systems, data accuracy

## Abstract

**Background:**

The endemic channel is a surveillance method that presents statistical indicators and visual representations of a disease’s historical dynamics. Its epidemic curve defines the central tendency of cases and their expected variation, providing 3 levels (ie, “safety,” “warning,” and “epidemic”) to assess the epidemiological status of a region. Parameters include the central tendency used as the epidemiological warning threshold (EWT), the size of the retrospective window, and the handling of previous outbreaks and zero values in data. The absence of clear guidelines for the selection of these parameters may compromise reproducibility and hinder outbreak definitions and responses for endemic diseases such as dengue.

**Objective:**

This study aimed to review the parameters of the endemic channel used for the definition and monitoring of dengue outbreaks in Colombia while quantitatively assessing the performance of the method.

**Methods:**

We reviewed institutional epidemiological bulletins in Colombia and quantitatively assessed the endemic channel in two main aspects: (1) the impact on the EWT of parameter selection regarding the retrospective data window, previous epidemic years handling, and zero-value handling, using a statistical framework; and (2) the endemic channel’s performance based on the windows of opportunity, outbreak detection capacity, and the ratio of warnings that correspond to actual outbreaks.

**Results:**

The endemic channel’s performance is higher as transmission increases due to more robust data that facilitate a timely detection of outliers, while lower-transmission areas show a sharper rise in cases when outbreaks are missed, indicating limited detection capacity. Reducing the retrospective data window improved metrics across all transmission profiles by 6.34% on average, while extending it decreased performance due to changes in detection capacity. There was no significant difference (*P* value >.01) in performance when data from epidemic years were included or excluded for municipalities with high or very high transmission levels. Instead of adding an entire unit, shifting the data by 0.001 prevents the estimation of null values for the EWT and thresholds and significantly improves performance across all transmission levels (*P* value <.01) by 23.07% on average.

**Conclusions:**

The endemic channel’s performance varies with the outbreak definition and the municipality’s transmission level. Encouraging an optimal retrospective window is challenging, as data are computed over the years. Nevertheless, the improved performance with shorter retrospective windows is likely due to reduced overlap in seasonal outbreaks. Shifting data by a limiting-to-zero value, instead of adding a complete unit, improves performance and can be easily integrated into existing surveillance templates. Windows of opportunity should be considered when selecting the parameter combination. Finally, reassessing outbreak definitions and method parameters underpinning surveillance tools is essential to ensure their validity and effectiveness, especially when used to inform early warning systems and public policies.

## Introduction

States and policymakers often use various methods to set dengue epidemic warning thresholds in endemic regions, resulting in different outcomes. One prevalent method is the endemic channel approach, which establishes thresholds based on recent data (often excluding epidemic years) by analyzing variability measures, such as the SD, and central tendency measures, such as the mean to, define an expected upper limit for cases (eg, mean plus 2 SDs) [1]. The endemic channel, introduced in 1999 by Bortman [2], exemplifies a surveillance tool that presents statistical metrics and a visual schematic of a disease’s historical dynamics in a specific region. It uses case data to construct an epidemic curve, showing cases over time and establishing the central tendency along with upper and lower limits where cases are expected to fluctuate. This method creates a graph with 3 thresholds, defining “safety,” “warning,” and “epidemic” zones, which are used to assess the region’s epidemiological situation based on current case counts.

The World Health Organization (WHO) reports an 8-fold increase in dengue cases over the last two decades, from approximately 505,000 cases in 2000 to more than 2.4 million in 2010 and 5.2 million in 2019 [3]. More recently, in 2024, the Americas region reported an unprecedented rise in dengue cases, exceeding 13 million notifications [4]. Colombia has been under epidemiological warning due to an increase in cases since the second quarter of 2022, prompting the intensification and strengthening of prevention, comprehensive care, surveillance, and control measures [5]. To support these activities, the endemic channel has been widely adopted in the Americas, including Colombia, the Dominican Republic, and Peru, to assess dengue surveillance and response [6]. The method, along with other outbreak definitions, such as deviation of bar charts and moving averages, has been evaluated by experts from the WHO’s Special Program for Research and Training in Tropical Diseases [6]. Their assessment highlights several key advantages of this approach: first, it provides a straightforward workflow for monitoring epidemic thresholds, allowing for the quick detection of outbreaks; second, its standardized outbreak definition promotes more transparent and consistent communication across diverse audiences, making messages easier to understand and compare; third, the endemic channel enables public health authorities to evaluate outbreak severity by examining factors, such as duration and total case numbers, thereby simplifying comparisons across different countries; and ultimately, it serves as a crucial tool for evaluating response effectiveness and informing decisions on whether to intensify or reduce intervention efforts, thereby enhancing outbreak management and control.

It is important to recall that the endemic channels focus on intraannual trends, calculating monthly or seasonal means to create a baseline, which simplifies operations but can lead to significant uncertainty [1]. As a result, the endemic channel can be quite uncertain, with outbreak thresholds being occasionally exceeded. These challenges have led to discussions and efforts to develop evidence-based models for dengue surveillance and outbreak management [6]. Methodologically, the endemic channel encompasses several parameters to be considered in its implementation. First, the central tendency measure used is the epidemiological warning threshold (EWT). Then, the size of the retrospective window (in years) for historical data is considered. Finally, the handling of previous outbreaks and zero values in the data is addressed. Owing to the direct influence of these parameters on algorithmic outcomes, an absence of clear institutional pipelines regarding their selection and underlying assumptions could hinder reproducibility and complicate the interpretation of results. This ambiguity can also foster experience-dependent usage and limit the comparability of sensitivity and specificity analyses across regions (eg, Bowman et al [7] and Schlesinger et al [8]) with differing transmission profiles, such as hypoendemic, endemic, and hyperendemic areas. Analytical pipelines implemented in open-access tools, such as the epiCo R package (R software developed by JDU, JM-T, and JO at Epiverse-TRACE) [9], could provide reproducible endemic channel workflows for other diseases or regions and promote the review of the mathematical assumptions embedded in current surveillance systems.

This paper examined how institutions define dengue outbreaks and evaluate the guidelines used to estimate the endemic channel at both national and departmental levels in Colombia. Specifically, we analyze the endemic channel’s outcomes by two main aspects: (1) testing how parameter choices—such as the retrospective data window, handling of previous epidemic years, and zero values affect results; and (2) proposing epidemiological metrics to assess the method’s performance based on the resulting windows of opportunity, outbreak detection capability, and epidemiological outcomes following a warning.

## Methods

### Overview

This study was conducted in 2 main stages. First, we performed a documentary analysis to characterize how endemic channels for dengue are constructed and used across Colombia. This involved a review of publicly available epidemiological reports at national and departmental levels. The second stage used previous results to establish an institutional baseline, understood as the set of parameters to use in the endemic channel, to assess experiments on the method using official linelist data.

### Ethical Considerations

The TRACE-LAC (Tools for Response, Analytics, and Control Enhancing of Epidemics in Latin America and the Caribbean) research project has a no risk classification according to the Ethics Committee of Universidad de los Andes, expresser in the Acta 1632 of 2022 and based on the Colombian resolutions 008430 of 1993 and 2378 of 2008. This work does not involve the manipulation of sensitive data or interaction with vulnerable communities or individuals, nor the performance of any experiment on subjects.

### Exploration of Institutional Reports and Outbreak Definitions

We first developed an analytical baseline to characterize how Colombia constructed and used endemic channels. To support this analysis, we retrieved the annual dengue reports from 2008 to 2022 from the National Institute of Health (Instituto Nacional de Salud [INS]) [10] and Health Secretariats’ websites, covering both the national and department (administrative division one) levels. From these reports, we aimed to identify the type of data and parameters used in constructing endemic channels and assess the consistency and availability of these information methods for constructing endemic channels nationwide. The compiled reports allowed us to establish parameters, such as the method, the retrospective window, zero-value handling, and the previous outbreak handling, which were subsequently used as a reference for the experiments applied to the data described below.

While analyses were outlined for Colombian municipalities with dengue transmission, we replicated the following protocol at departmental resolution to explore how endemic channel outcomes vary when aggregating data from settings with heterogeneous transmission levels. The stratification of transmission at the departmental level was assigned as the level to which most inhabitants within each department are exposed.

### Data Description

#### Epidemiological Data

**Figure 1.**
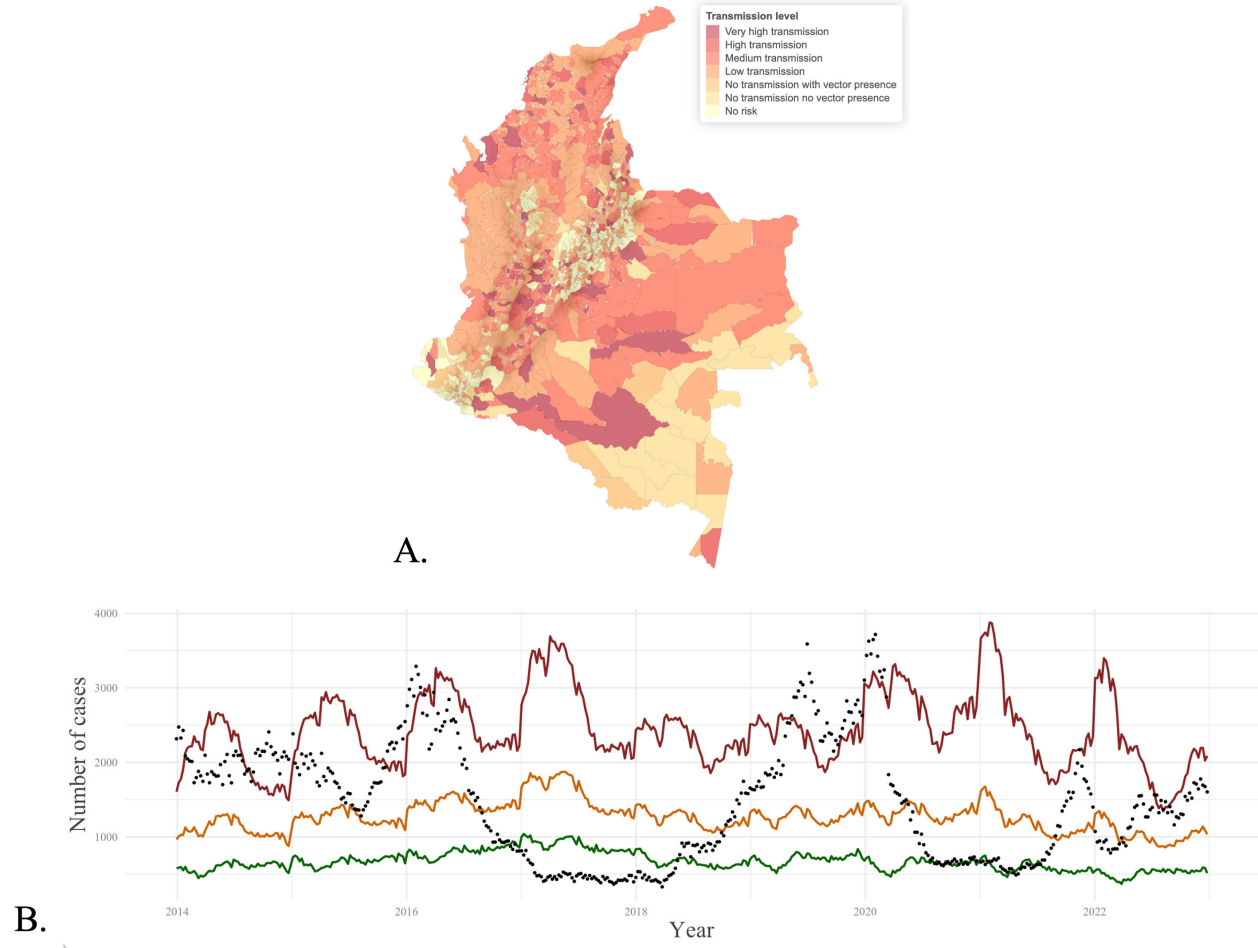
Epidemiological overview of dengue notification in Colombia. (**A**) Spatial distribution of dengue transmission levels for 2023, (**B**) Continuous endemic channels (from 2014 to 2022) at the national level. Black dots report weekly incidence; green, yellow, and red lines account for “safety,” “warning,” and “epidemic” levels, respectively.

Dengue linelist data between 2007 and 2022 in Colombia were downloaded from the National Surveillance System (SIVIGILA) website using the *sivirep* R package [11]. The dataset comprises anonymized individual case notifications, which were cleaned and harmonized by location codes. Cases were aggregated solely by municipalities or departments of occurrence and grouped by epidemiological weeks according to the symptom onset. Municipalities in Colombia are typically classified into 7 transmission levels by the Ministry of Health and the National Institute of Health [12]; the spatial distribution of the transmission levels and the national weekly incidence are shown in Figure 1A and B, respectively.

#### Estimation of the Endemic Channels

The endemic channels were estimated using the *epiCo* R package [9], which provides statistical analyses and visualizations of vector-borne disease outbreaks in Colombia. *epiCo* is a product of the TRACE-LAC Initiative and implements functions to estimate the expected number of cases as the EWT and the associated CIs used as safety and outbreak thresholds, as proposed by Bortman and explained in detail in Multimedia Appendix 1. We used the case data to calculate the endemic channel in the municipalities of Colombia. Figure 1B shows the endemic channels from 2014 to 2022 in a continuous graph.

### Endemic Channel Assessment Protocol

#### Overview

**Figure 2.**
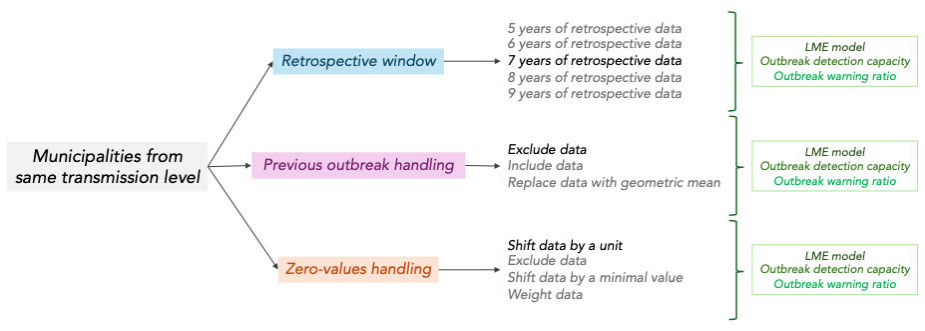
The quantitative assessment of the endemic channel parameters for dengue surveillance consisted of 3 experiments using the endemic channels of Colombian municipalities with the same transmission level, from 2014 to 2022. The changes in the epidemiological warning threshold of each experiment were evaluated with a linear mixed-effects (LME) model. The outbreak detection capacity and the warning ratio metric assess the effectiveness of the outbreak warning; both metrics contribute to the overall performance evaluation of the endemic channels. In each experiment, parameters shown in bold text represent the resulting baselines used for comparison.

We quantitatively assessed the impact of the endemic channel parameters, focusing on 2 key aspects: their effect on the EWT and their effect on the endemic channel’s performance. Figure 2 outlines the implemented protocol. We tested parameter configurations using municipalities grouped by similar transmission levels. Three parameters of the endemic channel method were systematically assessed: the retrospective window, the handling of previous outbreaks, and the handling of zero values. The process was conducted separately for the sake of interpretation, assuming that it is not possible for a region to exhibit exceptional outbreaks and systematic zero cases within its historical records.

The retrospective window, defined as the number of years of historical data used for threshold estimation, was tested, ranging from 5 to 9 years. To handle previous outbreaks within the retrospective window, three strategies were considered: (1) excluding outbreak periods, (2) including them as-is, or (3) replacing them with the geometric mean of the remaining dataset. To evaluate the effect of zero-value handling strategies, we (1) shifted the number of cases by a unit to avoid zero values as stated by Bortman [1], prior to the EWT calculation, to later be subtracted; (2) excluded data with zero values from the computation; (3) shifted data by a minimal value (0.001 for the purpose of this study) prior to EWT calculation to later be subtracted, as an alternative approach to Bortman; and (4) weighted the estimations by the fraction of zero values present in the data; these last 2 approaches are inspired by de la Cruz and Kreft’s [13] and Habib’s [14] approaches to estimating the geometric mean of a dataset with zeros. Each parameter combination was then evaluated using a linear mixed-effects (LME) model and a composite performance metric for outbreak detection capacity and warning ratio.

#### LME Model on the EWT

The following analysis evaluates whether the experiments shown in Figure 2 produce a significant increase or decrease in the EWT. The epidemiological interpretation of the assessment is that changes in the EWT ultimately define the “warning” threshold level, which greatly influences the triggering of the method and subsequent performance evaluations. To ensure a valid evaluation, the analysis recognizes that observations within the same municipality are not independent but influenced by underlying trends over time.

Therefore, we applied an LME model with the EWT as the response variable and each experiment depicted in Figure 2 as a fixed effect. We also incorporated a random effect structure to control for within-group correlations in the observations of the same municipality and date (Equations 3 to 5).


Equation 1 $$EWT_{ij}=\beta_{0}+\beta_{1}Experiment_{i}+u_{j\left(i\right)}+\epsilon_{ij}$$

Equation 2 $$u_{j\left(i\right)}∼N\left(0,\sigma_{u}^{2}\right)$$

Equation 3 $$\epsilon_{ij}∼N\left(0,\sigma^{2}\right)$$

where *u*_*j*(*i*)_ is the random intercept for the group *j*(*i*), that is, municipality *j* with observation at time *i*, *σ*_*u*_^2^ represents the variance of this intercept, and *t* describe the variability across the municipalities The model coefficients’ analysis focused on whether the EWT was significantly impacted (*P* value <.01) by each experiment, captured by *β*_1_, and the percentage of change that this coefficient induces.

#### Epidemic Features and the Endemic Channel Performance

**Figure 3.**
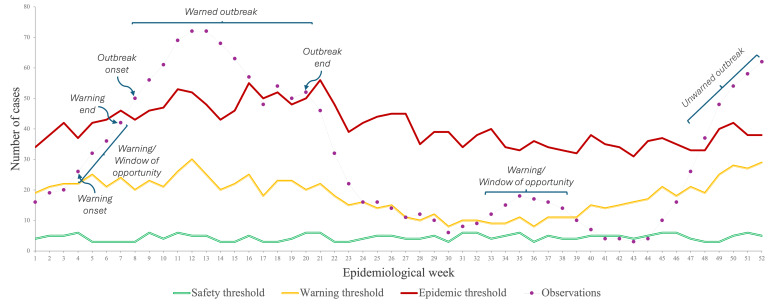
Graphical representation of the epidemic features based on the Joint External Communication [15] from the Ministry of Health and the National Institute of Health. Two warnings at weeks 4 and 33 are shown: the first one resulted in an outbreak with a window of opportunity of 4 weeks, while during the second warning, the increase in cases was controlled in a window of 6 weeks. Cases from weeks 18, 19, and 20 were not classified as a new outbreak because the previous warning did not last 2 or more weeks. Similarly, there was no triggered warning at weeks 25, 28, and 47 because cases were over the epidemiological warning threshold for only 1 week, the last scenario leading to an unwarned outbreak.

We developed an algorithm that calculates several epidemic features, such as the outbreak’s onset (the first of 3 or more consecutive weeks when cases are above the upper limit after being at least 2 weeks below it), the total number of warnings (the first of 2 or more consecutive weeks when cases are between the EWT and the upper limit), the number of warnings that resulted in an outbreak, the number of outbreaks that occurred without triggering a warning, and the windows of opportunity. A more detailed definition of these features can be found in Table S1 in Multimedia Appendix 1 and is exemplified in Figure 3.

On the basis of these epidemic features, we propose 3 metrics to assess the endemic channel methodology: the outbreak detection capacity, the outbreak warning ratio, and the endemic channel performance. Metrics are described in equations 6, 7, and 8. To weigh the impact of outbreak detection capacity and the outbreak warning ratio equally, the parameter is set to 0.5.


Equation 4 $$Outbreak\;detection\;capacity\;=\;\frac{Number\;of\;warned\;outbreaks}{Total\;number\;of\;outbreaks}$$

Equation 5 $$Outbreak\;warning\;ratio\;=\;\frac{Number\;of\;warned\;outbreaks}{Number\;of\;triggered\;warnings}$$

Equation 6 $$\begin{array}{l} Endemic\;channel\;performance=\;\alpha \cdot Outbreak\;detection\;capacity\;+\\ \left(1-\alpha \right)\cdot Outbreak\;warning\;ratio \end{array}$$

Although we did not assess the endemic channel as a forecasting tool, we aimed to gain insight into the window of opportunity that the method offers in the studied context. The window, then, was defined for all experiments as the delay in weeks between a warning onset (first of 2 or more consecutive weeks when cases are between the EWT and the upper limit) and an outbreak onset (first of 3 or more successive weeks when cases are above the upper limit after being at least 2 weeks below it). Significance in epidemic features was assessed with a mixed-effects ANOVA for a *P* value <.01.

## Results

### Overview

Our exploration of institutional reports and outbreak definitions did not show a standardized set of parameters; however, the review of national surveillance guidelines and Bortman’s original reference informed the selection of the following baseline parameters for evaluating endemic channel outcomes: (1) method: geometric mean; (2) zero-value handling: addition of one unit to the data; (3) outlier year: 2019; (4) outlier handling: exclusion; and (5) retrospective window: 7 years. Table S2 in Multimedia Appendix 1 displays the findings on the method used to estimate the endemic channel for each year, the number of years to consider a retrospective window, and the handling of epidemic years. None of the reports displayed the numerical values of the EWT or the epidemic thresholds.

The same previous baseline was used in the assessment at the departmental level due to the limited number of epidemiological bulletins at this administrative level that reported the used methodology and the heterogeneity in the selection of its parameters when documented. For example, in 2022, from the 28 departments with dengue transmission, only 12 displayed an endemic channel graph, 8 of these documented the used central tendency measure (12.5% used the mean, 12.5% used the geometric mean, 37.5% used the median, and 37.5% reported using more than one method during the year); and, in the same year, the window size was reported only in 16.7% of the departments, varying between 5 and 9 years of data. Tables S3 and S4 in Multimedia Appendix 1 contain the resulting classification of dengue transmission at the departmental level and the corresponding parameters, respectively.

The following subsections detail the results by parameter and by transmission level and describe the behaviors of the windows of opportunity and spatial aggregation when analyzed at the departmental level. As noted in the methodology, only one parameter was varied at a time relative to the baseline, and therefore, parameter combinations were not assessed. Consequently, it cannot be determined whether combining the best-performing settings for each parameter would yield superior overall results.

### Assessment of the Retrospective Window

**Figure 4.**
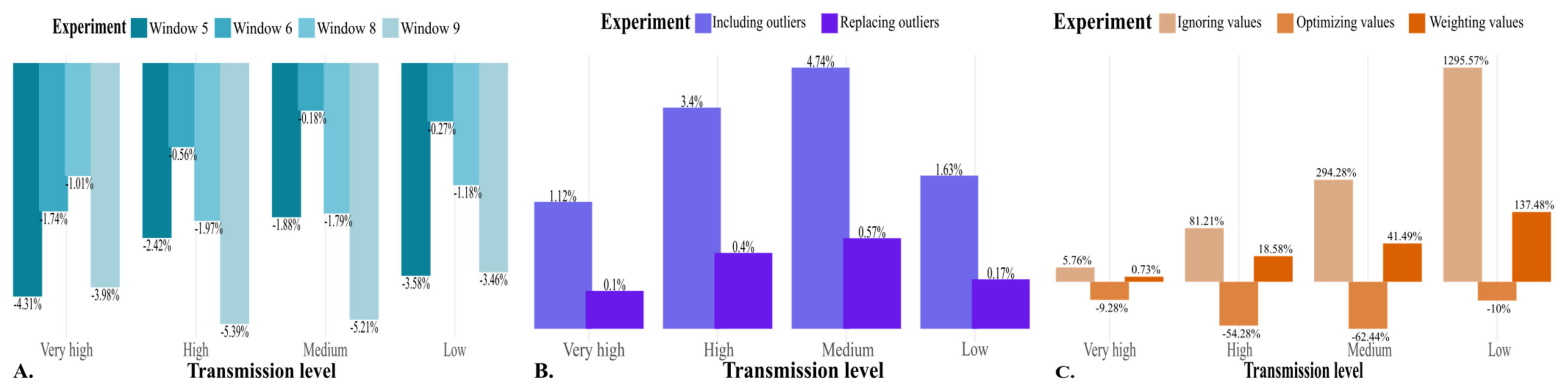
Endemic channel’s parameters effect on the epidemiological warning threshold: a linear mixed-effects model analysis. (**A**) Retrospective data windows experiment, (**B**) previous outbreaks handling experiment, (**C**) zero-value handling experiment. Percentages displayed as the logarithm of the value. Detailed data can be accessed in Tables S5, S7, and S9 in Multimedia Appendix 1.

**Figure 5.**
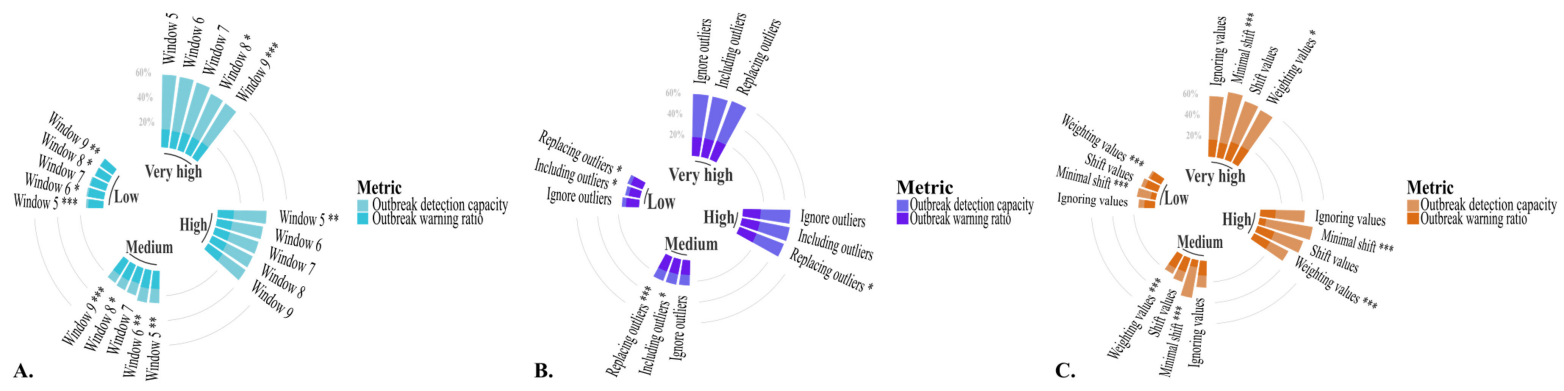
Endemic channel’s parameters effect on the endemic channel performance: a composite metric including the number of warned outbreaks over the total number of outbreaks (outbreak detection capacity) and the number of warned outbreaks over the total number of triggered warnings (outbreak warning ratio). (**A**) Retrospective windows experiment, (**B**) previous outbreaks handling experiment, (**C**) zero-value handling experiment. Performance is proposed as the contribution of the detection capacity (light colors) and the outbreak warning ratio (dark colors). Detailed data can be accessed in Tables S6, S8, and S10 in Multimedia Appendix 1. Significance levels: *P<.05, **P<.01, ***P<.001.

On the basis of the 7-year retrospective data window, the EWT decreased relative to the baseline in all cases. The reduction was particularly pronounced when the extremes of the data window were assessed—specifically, 5 and 9 years of retrospective data—as illustrated in Table S5 in Multimedia Appendix 1 and Figure 4A. The only exception was the 6-year retrospective data window for municipalities with low and medium transmission levels, which did not significantly change the EWT. Figure 5A depicts the influence of retrospective window size on the performance of the endemic channel. Across all transmission levels, performance declined as the retrospective window increased, primarily due to a reduction in outbreak detection capacity. Notably, municipalities with higher transmission levels demonstrated better endemic channel performance overall. Furthermore, municipalities with low and medium dengue transmission were the most substantially affected by variations in the retrospective data window, highlighting the sensitivity of these settings to the parameters studied.

### Assessment of How Previous Outbreaks Are Handled

For both treatments, the EWT increased compared to the baseline strategy of ignoring previous outbreaks, with a greater increase observed when data from prior outbreaks were incorporated, as shown in Table S7 in Multimedia Appendix 1 and Figure 4B. The impact of handling previous outbreaks on endemic channel performance is illustrated in Figure 5B. In low-transmission municipalities, the highest performance was achieved when ignoring data from previous outbreak years. In contrast, replacing outbreak data with a geometric mean resulted in better performance for medium and very-high-transmission municipalities. For high transmission municipalities, the best performance was observed when data from previous outbreaks were included. Overall, as transmission levels increase, the performance of the endemic channel improved across different handling methods.

### Effect of Zero-Value Handling

The minimal shift was the only strategy that decreased the EWT compared to the baseline (shifting the data by a unit), as seen in Table S9 in Multimedia Appendix 1 and Figure 4C. The percentage of change was noticeably greater in low- and medium-transmission municipalities, as expected given their higher frequency of zero values.

The impact of zero-value handling strategies on endemic channel performance is depicted in Figure 5C. Metrics for the ignored-value and weighted-value strategies are presented for municipalities where we could estimate these metrics; however, the endemic channel consistently failed when these strategies were applied in cases where retrospective data lacked sufficient nonzero values. Across all transmission levels, performance significantly improved when using the minimal shift strategy. Notably, higher transmission levels corresponded with better endemic channel performance. Moreover, on average, all municipalities, regardless of their dengue transmission levels, were significantly influenced by applying the minimal shift and weighting strategies.

### Windows of Opportunity Behavior

**Figure 6.**
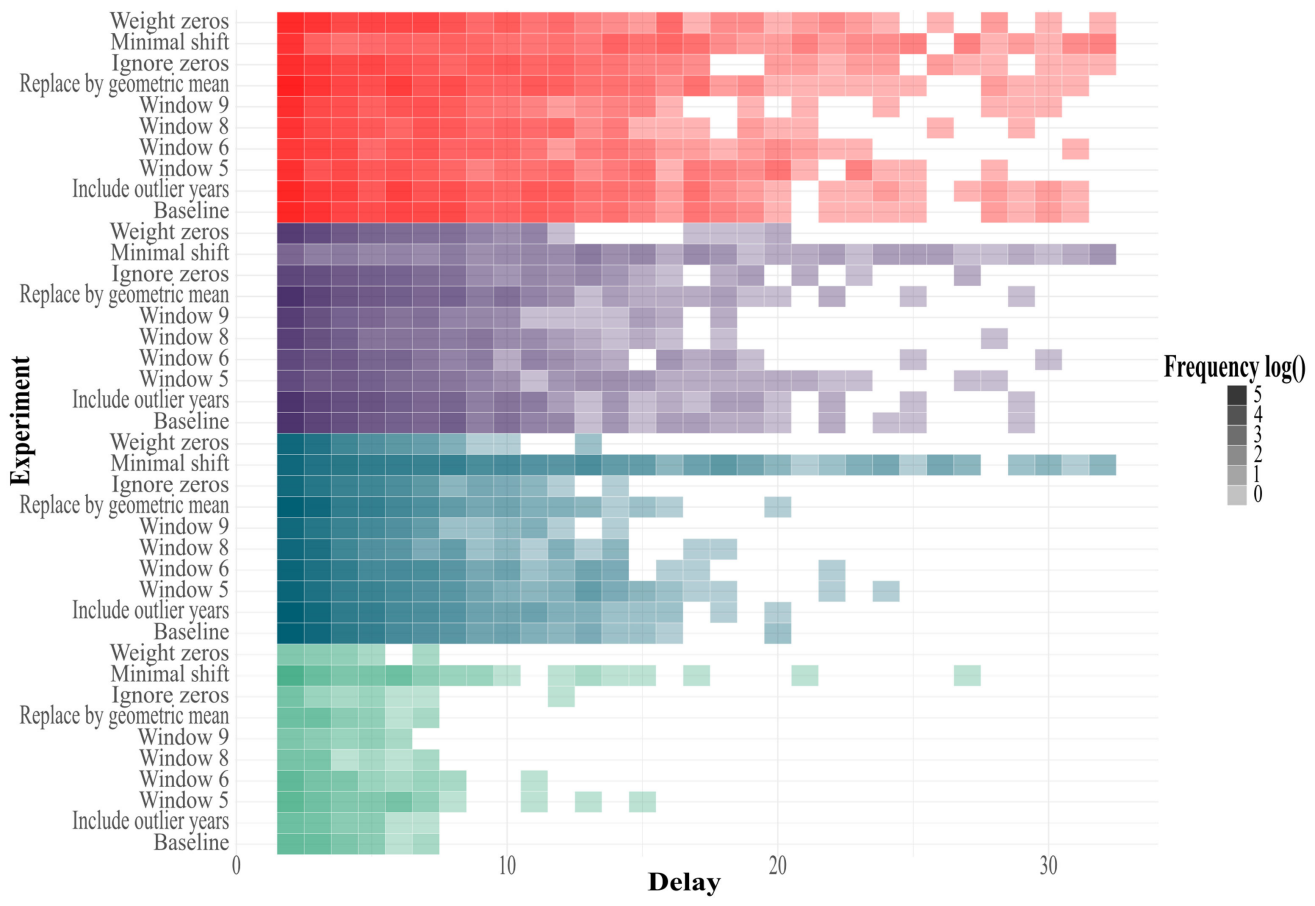
Windows of opportunity distributions across experiments on very high (red), high- (violet), medium- (turquoise), and low-transmission (mint) municipalities. Color intensity is the logarithm of the frequency of observed delays, baselines refer to the institutional protocol, and the figure is shown up to a 32-week delay. Over-a-year outliers were found in municipalities with very high, high, and medium transmission.

We identified that the 2-week delay is the shortest and most predominant window of opportunity in all transmission levels and experiments, as expected from the definition of the outbreak onset used in this study. Figure 6 displays the distribution of the windows of opportunity for all experiments across the different transmission levels. Neither the endemic channel nor our predictive assessment considered the magnitude or duration of the outbreak following the warning or the intraannual trend in case numbers.

In general, at the municipality level, the experiments allowed us to identify 2 settings that consistently improved the performance of endemic channels across all transmission levels: reducing the retrospective window relative to the baseline and applying a minimal shift to data (0.001) instead of adding a complete unit to handle zero values. In contrast, the management of previous outbreaks produced heterogeneous effects across transmission levels. When evaluated at departmental scales, the endemic channel significantly outperformed municipal results in all experiments and all transmission profiles, as seen in Tables S11, S12, and S13 in Multimedia Appendix 1. For instance, the baseline protocol showed a performance approximately 4 times greater for departments with low transmission, 2.3 times greater for those with medium transmission, 1.4 times greater in high transmission settings, and 1.2 times greater for departments with very-high transmission, compared to the performance at the municipality level. In contrast to the experiments at the municipal level, the effect of the retrospective window size, previous outbreak handling, and zero-value handling did not exhibit a statistically significant difference compared to the baseline, leading to similar conclusive insights and recommendations provided in this study.

## Discussion

### Principal Findings

Our assessment of the endemic channel parameters shows that reducing the retrospective data window improved metrics across all transmission profiles by 6.34% on average, while extending it decreased performance due to changes in detection capacity. It also highlights that the additional step of excluding outbreak years, such as 2019, in the estimation of the EWT does not significantly affect the method’s performance in municipalities with very high and high transmission but produces significant differences in medium- and low-transmission units, where performance changed by approximately +1.48% and –14.82%, respectively. Experiments on handling zero values demonstrated that adding a small number to observed cases (such as 0.001) not only prevents null estimates of epidemic thresholds but also improves overall performance by an average of 23.07% across all transmission levels. An analysis of the window of opportunity at the baseline showed that its duration varies with transmission intensity; with an average of 8 weeks for very high, 5 weeks for high, 4 weeks for medium, and 3 weeks for low-transmission municipalities, reflecting that the sharpness of windows as transmission decays may make the anticipation of outbreaks difficult.

The use of metrics such as the outbreak detection capacity or the endemic channel performance proposed in this study faces challenges comparable to the definition of an outbreak. For example, Brady et al [16] modeled 5 candidate definitions of an outbreak (recent mean, monthly mean, moving mean, cumulative mean, and fixed incidence threshold) and found that resulting outbreaks varied significantly in terms of frequency, duration, and impact. Additionally, outbreak definitions vary in timing, affecting their suitability for early intervention. Likewise, our results show that the behavior of the endemic channel performance differs according to the selected outbreak definition (tuned by the parameters used in the method and the definitions of the epidemic features) and the transmission context. In addition, implementing performance metrics on outbreak detection methods that exhibit different definitions hinders the comparison of epidemiological scenarios within regions or periods. For example, previous assessments on epidemic thresholds report a limited sensitivity of 40% (accounting for warnings that lead to a significant increase in cases) and a specificity of 90% in Puerto Rico when using 2 SDs as CIs [17]. In contrast, a sensitivity of 66% in Brazil was reported when using 1 SD [18]. These assessments differ from the endemic performance proposed in this study because they do not account for diverse definitions of outbreaks based on other parameters of the surveillance method besides the SD as a threshold size.

To the best of our knowledge, no prior study has examined the effect of retrospective window size, previous outbreak handling, and alternative strategies to handle zero values on the outcomes generated by the endemic channel method. It is challenging to encourage a shorter window, as the selection of retrospective data is defined in year units and hinders the ability to identify an optimal window size. Excluding specific years from the retrospective data could lead to inconsistent usage of the endemic channel, primarily because the selection uses the same criteria once the potential epidemic year has already occurred. In addition, our analyses support the decision to include previous outbreak data with minimal impact on the number of triggered warnings. When evaluating the different experiments to handle the occurrence of zero values in the retrospective data, we identified that the selection is restricted to the shifting strategies in the scenario where all data are zero. Therefore, shifting data with a minimal value is equally feasible to implement in existing surveillance templates in official institutions. It is important to recall that the minimal shift strategy does lead to a significant reduction in the EWT, especially in municipalities with high and medium transmission. This will generate more warnings, and local authorities may review their response protocols to avoid exhaustion of surveillance and control programs. This behavior is better understood when the delay between the warning signal and the outbreak onset is considered, as the following sections in the study do. Given the nature of surveillance data, it is challenging to evaluate if the persistence of the warning is caused by a slow-paced transmission or a mitigated outbreak, for example. Ultimately, for municipalities with very high, high, and medium transmission, the window of opportunity provided more information regarding the amount of time that the system is in a warning status than regarding the timeliness and specificity of the endemic channel to trigger a warning for nonseasonal and significant outbreaks. On the other hand, the low-transmission municipalities display short periods between warnings and outbreaks, reflecting their outbreaks’ sharp, intermittent, and difficult-to-anticipate behavior. The temporal criteria in the onset definition, while needed to address that the threshold lines are often surpassed briefly and intermittently [6], condition the analysis of the timeliness of the endemic channel warning, as the minimum delay is policy rather than data driven.

### Study Limitations

The results presented in this study rely on the assumption of a constant guideline for constructing endemic channels, which we subsequently used as baselines regardless of spatial or temporal setting. To enable analyses across all municipalities and years, we did not validate the warnings triggered by these baselines or the experiments against public or private records of health responses. If outbreak definitions were influenced by information beyond reported case data and its expected epidemiological limits, our approach does not capture such adjustments, as it does not account for external variables.

Similarly, the performance metrics used to assess the outcomes of the endemic channel were not compared with other methodologies or with external variables that might better reflect the true epidemiological situation of the regions. Consequently, there is limited certainty that governmental institutions would classify as outbreaks the same events we define as such. Furthermore, our interpretation of the metrics assumes an ideal performance of maximizing the detection of actual outbreaks while minimizing the number of triggered warnings, which may also be biased or constrained from the perspective of a public health officer. This absence of a ground truth dataset, combined with the statistical limitations inherent to the endemic channel, led us to avoid forecasting evaluations or other assessments that may exceed the practical uses of the surveillance method.

Finally, the statistical aggregation of our experiments relies exclusively on the Ministry of Health classification of dengue transmission levels. This stratification was not contrasted with additional approaches and may hinder population and disease dynamics that shape the expected variation of cases and the occurrence of outbreaks. The heterogeneity of our results and their dependency on the transmission level prevent us from recommending a single guideline; instead, they support the need to evaluate the appropriateness of the endemic channel in settings with low disease incidence.

### Conclusions

Our results show that the endemic channel’s performance significantly varies according to the outbreak definition and transmission level. The metrics exhibit better performance as the transmission level increases, likely due to (1) more robust retrospective data for high transmission settings, which supports the identification of transmission outliers; and (2) a sharper increase in cases in municipalities with lower transmission when outbreaks occur that are not detected by the endemic channel on time (ie, low outbreak detection capacity in these settings). Therefore, the timeliness of the warnings needs to be considered, especially in departments with a considerable proportion of low- and medium-transmission municipalities because notification aggregation can obscure the occurrence of incipient outbreaks in these areas.

Concerns about the definition and usage of outbreak thresholds have been previously expressed by the WHO’s Special Program for Research and Training in Tropical Diseases [6,19]. In their study, the committee gathered evidence of methods’ weaknesses, such as health officers may mislead the outbreak threshold as a “warning sign” rather than an already triggered epidemic; a satisfactory algorithm for recognizing past aberrations has yet to be developed [20]; previous outbreaks may lead to an exaggerated increase in thresholds; seasonal outbreaks may differ in frequency from the fixed retrospective window; and, when geographical units are small, the variation in cases tends to increase, potentially resulting in significant oscillations. This study provides insights into some of these concerns and aims to promote the frequent assessment of methodologies and definitions used in institutional outbreak surveillance, especially in the increasing interest in automated surveillance systems.

Given the relevance of the disease in the region, it is crucial to develop and implement tools to predict possible outbreaks at different geographical scales. In this context, the WHO Global Arbovirus Initiative, for example, has emphasized the importance of innovative tools to promote integration of social and mathematical sciences with public health and include climate and environmental context in early warning and forecasting systems [21]. The initiative also highlights methodological gaps when integrating surveillance systems, field and/or laboratory confirmation, and epidemiological investigations. For example, whereas a predictive system is a deterministic or probabilistic model that uses historical data and identified patterns to infer future values [22], tools such as the endemic channel do not meet this definition, as it is a descriptive statistical method that does not make any inference of future cases. Therefore, to use the endemic channel as a forecasting system, it is necessary to incorporate complementary evidence from other tools, such as stochastic methods [23] or probabilistic models [24]. Additionally, as noted in the various methodologies, it is essential to consider the multiple factors that can affect the presence and biology of the vector, such as climate variability, water storage, population density, and socioeconomic conditions that have been shown to impact the disease behavior [25]. An early literature review of dengue epidemiological surveillance found that, although 46 of the 56 major dengue-endemic countries had dengue surveillance systems in place, only 4 had systems robust enough for epidemic prediction [26,27], and that no plan provided for the use of climate data to predict an outbreak [28]. For this reason, it is advisable not to use the endemic channel as the only source of information for decision-making but to consider other tools that incorporate these factors.

While the endemic channel is a methodology that overlooks the pathogen’s transmission dynamics and local contexts, the assessment of parameters for other epidemiological events should be carefully considered. For example, we encountered challenges when defining a consensus of the institutional baselines after reviewing the guidelines and definitions published by official institutions in Colombia. Therefore, caution should be exercised when extrapolating our insights from this study to other contexts and diseases. With this study, we extend an invitation to frequently question and review the mathematical assumptions embedded in current surveillance methods to ensure their correct use and interpretation, especially when their results are used as warning systems in public health response policies.

### Policy Recommendations

Governmental institutions may benefit from public communication of their methodology and parameters when constructing an endemic channel. The usage of the method, as analyzed through the epidemiological bulletins, is heterogeneous across regions and years. Notably, it is often difficult to identify the underlying assumptions and data workflows used in its development, which hinders the ability to replicate results, to establish a baseline, and to compare the epidemiological situation across municipalities or departments.

Including longitudinal (intraannual) analysis in a surveillance methodology, such as the endemic channel, is necessary to strengthen the tool’s capacity to account for dengue transmission dynamics and weight notification data according to its recency. Meanwhile, we observed that the superior performance of shorter windows could be explained by the lower overlap of seasonal outbreaks, which occur approximately every 3 years in Colombia. Encouraging an optimal retrospective window is challenging, as data are computed by years, but the endemic channel for dengue surveillance in other regions may improve its performance if its retrospective data window is synchronized with the regional seasonality of the disease.

Alternative analyses to account for seasonal outbreaks should be incorporated when evaluating the epidemiological situation of a region. While removing previous epidemic years from the retrospective data helps mitigate outlier effects on the endemic channel, this practice did not yield a systematic improvement in performance metrics. Despite statistically significant differences in medium- and low-transmission municipalities, we found that this approach compromises the method’s reproducibility in higher transmission settings, where its consistency may be more critical.

The addition of a small value to observed cases before the EWT estimation, rather than a complete unit, can be easily implemented for the benefit of the endemic channel’s performance metrics. Although this change can increase the number of warnings, additional surveillance activities and tools can enlighten the correct outbreak response in these settings.

## Supplementary material

10.2196/79914 Multimedia Appendix 1 Endemic channel algorithm, the exploration of institutional reports and outbreak definitions, and assessment results.

## Abbreviations

EWT: epidemiological warning threshold
GAIDeT: Generative Artificial Intelligence Delegation Taxonomy
INS: Instituto Nacional de Salud
LME: linear mixed-effects
TRACE-LAC: Tools for Response, Analytics, and Control Enhancing of Epidemics in Latin America and the Caribbean
WHO: World Health Organization
