# Supplementary material for: Endemic Channel Parametrization in Dengue Surveillance: Methodological Assessment of Retrospective Windows, Outbreak Trends, and Zero-Case Periods in Colombia
Source: JMIR Public Health Surveill. 2026 May 8;12:e79914. doi: 10.2196/79914 (PMC13155432; doi:10.2196/79914)
Supplement: Multimedia Appendix 1 [file publichealth-v12-e79914-s001.docx]

Supplementary Material:

Data description

*Estimation of the endemic channels:*

The endemic channel algorithm implemented in *epiCo* is expressed mathematically in Equations 1 and 2:

${EWT}_{w,y+1}=exp\left( \frac{1}{n}\sum_{j=0}^{n} log(C_{w,y-j}) \right)$ Equation 1

$CI95\% ={EWT}_{w,y+1} \pm tDE/\surd n$ Equation 2

Where $C_{w,y}$ represents the expected number of cases at week $w$ and year $y$. It is noticeable that the EWT is the geometric mean of the reported cases for the week of interest during previous $n$ years, and that the confidence interval is calculated using a t-student distribution.

Endemic channel assessment protocol

*Epidemic features and the endemic channel performance:*

Supplementary Table 1. Definition of epidemic features

| Feature | Definition |
| --- | --- |
| Outbreak onset | First of three (or more) consecutive weeks when cases are above the upper limit after being at least two weeks below it |
| Outbreak end | Last week before cases drop below the upper limit for at least two weeks |
| Warning onset | First of two (or more) consecutive weeks when cases are between the EWT and the upper limit |
| Warning end | Last week before cases drop decrease below the EWT or increase over the upper limit |
| Warned outbreak | An outbreak onset that is preceded by a warning |
| Un-warned outbreak | An outbreak onset that is not preceded by a warning |
| Window of opportunity | Delay in weeks between a warning and an outbreak onset |

Exploration of Institutional reports and outbreak definitions

Supplementary Table 2. Endemic channel parameters for dengue in Colombia reported by the INS

| Year | Epidemiological Warning Threshold | Retrospective window size | Ignored epidemic year |
| --- | --- | --- | --- |
| 2008 | Median | Not reported | Not reported |
| 2009 |  |  |  |
| 2010 |  |  |  |
| 2011 |  |  |  |
| 2012 |  |  |  |
| 2013 |  | 4 | None |
| 2014 |  | 5 |  |
| 2015 |  | 7 |  |
| 2016 |  | 8 |  |
| 2017 | Geometric mean | 6 |  |
| 2018 |  | 7 |  |
| 2019 |  | 8 |  |
| 2020 |  | 7 | 2019 |
| 2021 |  | 7 |  |
| 2022 |  | 8 |  |

Supplementary Table 3. Proposed stratification for dengue transmission at departmental scale in Colombia. Transmission level is assigned according to the level at which most of the inhabitants are exposed.

| Department | Total population | Low transmission | Mild transmission | High transmission | Very-high transmission | Assigned transmission |
| --- | --- | --- | --- | --- | --- | --- |
| Amazonas | 82,068 | 17.93% | 0.00% | 63.37% | 0.00% | High |
| Antioquia | 6,887,306 | 10.66% | 16.77% | 5.66% | 55.07% | Very High |
| Arauca | 304,978 | 3.08% | 42.60% | 54.32% | 0.00% | High |
| Archipiélago de San Andrés, Providencia y Santa Catalina | 65,228 | 9.83% | 90.17% | 0.00% | 0.00% | Mild |
| Atlántico | 2,804,025 | 5.36% | 12.13% | 11.27% | 71.24% | Very-high |
| Bogotá, D.C. | 7,901,653 | 0.00% | 0.00% | 0.00% | 0.00% | No risk |
| Bolívar | 2,236,603 | 23.63% | 15.74% | 3.71% | 56.92% | Very-high |
| Boyacá | 1,259,601 | 13.02% | 4.50% | 3.88% | 0.00% | No risk |
| Caldas | 1,036,455 | 34.85% | 56.13% | 7.27% | 0.00% | Mild |
| Caquetá | 419,275 | 38.64% | 6.40% | 0.00% | 54.96% | Very-high |
| Casanare | 442,068 | 9.04% | 17.93% | 22.68% | 49.57% | Very-high |
| Cauca | 1,516,018 | 72.00% | 16.74% | 0.00% | 0.00% | Low |
| Cesar | 1,341,697 | 0.35% | 33.53% | 15.82% | 50.31% | Very-high |
| Chocó | 553,519 | 70.39% | 29.61% | 0.00% | 0.00% | Low |
| Córdoba | 1,856,496 | 10.75% | 37.76% | 23.85% | 27.63% | Mild |
| Cundinamarca | 3,478,323 | 8.26% | 6.48% | 6.38% | 5.46% | No risk |
| Guainía | 52,061 | 20.92% | 67.16% | 0.00% | 0.00% | Mild |
| Guaviare | 90,357 | 0.00% | 27.45% | 0.00% | 64.42% | Very-high |
| Huila | 1,140,932 | 9.69% | 21.46% | 16.04% | 50.52% | Very-high |
| La Guajira | 1,002,394 | 13.53% | 60.44% | 26.03% | 0.00% | Mild |
| Magdalena | 1,463,427 | 19.31% | 38.00% | 4.94% | 37.75% | Mild |
| Meta | 1,080,706 | 0.67% | 16.27% | 13.38% | 69.40% | Very-high |
| Nariño | 1,629,181 | 21.58% | 1.10% | 0.00% | 15.79% | No risk |
| Norte de Santander | 1,651,278 | 8.83% | 8.66% | 8.74% | 68.81% | Very-high |
| Putumayo | 369,064 | 0.00% | 28.29% | 23.12% | 39.15% | Very-high |
| Quindío | 569,569 | 6.75% | 6.27% | 32.11% | 54.88% | Very-high |
| Risaralda | 977,829 | 20.94% | 3.23% | 26.20% | 49.64% | Very-high |
| Santander | 2,324,090 | 10.43% | 8.19% | 7.77% | 69.48% | Very-high |
| Sucre | 972,350 | 13.50% | 44.01% | 11.52% | 30.97% | Mild |
| Tolima | 1,346,935 | 3.81% | 18.96% | 11.97% | 61.68% | Very-high |
| Valle del Cauca | 4,589,278 | 4.53% | 10.89% | 16.36% | 68.22% | Very-high |
| Vaupés | 48,932 | 73.24% | 0.00% | 0.00% | 0.00% | Low |
| Vichada | 115,778 | 3.71% | 96.29% | 0.00% | 0.00% | Mild |

Supplementary Table 4. Findings from institutional websites. Heterogeneity of parameters supports our usage of a single baseline.

| Department | Link | 2022 | 2021 | 2020 | 2019 |
| --- | --- | --- | --- | --- | --- |
| Amazonas | - | - | - | - | - |
| Antioquia | <https://dssa.gov.co/boletin-epidemiologica-de-antioquia-bea> | 23th-26th week, endemic channel graph presenting: alert, safety and success zone, and cases. | - | - | - |
| Arauca | - | - | - | - | - |
| Archipiélago  San Andrés, Providencia y Santa Catalina | <https://sanandres.gov.co/index.php/publicaciones> | - | - | - | - |
| Atlántico | [https://www.atlantico.gov.co/index.php/boletin-epidemiologico](https://nam10.safelinks.protection.outlook.com/?url=https%3A%2F%2Fwww.atlantico.gov.co%2Findex.php%2Fboletin-epidemiologico&data=05%7C02%7Cmc.tavera%40uniandes.edu.co%7Ce8e2ba40805743388a7508dd46f27a30%7Cfabd047cff48492a8bbb8f98b9fb9cca%7C0%7C0%7C638744729479713066%7CUnknown%7CTWFpbGZsb3d8eyJFbXB0eU1hcGkiOnRydWUsIlYiOiIwLjAuMDAwMCIsIlAiOiJXaW4zMiIsIkFOIjoiTWFpbCIsIldUIjoyfQ%3D%3D%7C0%7C%7C%7C&sdata=mfogPnAO10RNBM2wXQ80V4hAvy7m6tjxaHMnm6kDNJU%3D&reserved=0) | 21st-24th week, endemic channel graph presenting: upper and lower limit, and geometric mean.  Other epidemiological weeks:  Endemic channel graph presenting: lower confidence interval and cases mean. | Endemic channel graph presenting: lower confidence interval and cases mean. | - | - |
| Bolívar | - | - | - | - | - |
| Boyaca | [https://www.boyaca.gov.co/boletines-epidemiologicos/](https://nam10.safelinks.protection.outlook.com/?url=https%3A%2F%2Fwww.boyaca.gov.co%2Fboletines-epidemiologicos%2F&data=05%7C02%7Cmc.tavera%40uniandes.edu.co%7Ce8e2ba40805743388a7508dd46f27a30%7Cfabd047cff48492a8bbb8f98b9fb9cca%7C0%7C0%7C638744729479727974%7CUnknown%7CTWFpbGZsb3d8eyJFbXB0eU1hcGkiOnRydWUsIlYiOiIwLjAuMDAwMCIsIlAiOiJXaW4zMiIsIkFOIjoiTWFpbCIsIldUIjoyfQ%3D%3D%7C0%7C%7C%7C&sdata=dqB%2BzyyZQ21pa58lFnoMl0im0h5SahJWFu0RUPNWYEk%3D&reserved=0)  [https://www.datos.gov.co/Salud-y-Protecci-n-Social/EVENTOS-NOTIFICADOS-AL-SISTEMA-NACIONAL-DE-VIGILAN/shn8-kdew](https://nam10.safelinks.protection.outlook.com/?url=https%3A%2F%2Fwww.datos.gov.co%2FSalud-y-Protecci-n-Social%2FEVENTOS-NOTIFICADOS-AL-SISTEMA-NACIONAL-DE-VIGILAN%2Fshn8-kdew&data=05%7C02%7Cmc.tavera%40uniandes.edu.co%7Ce8e2ba40805743388a7508dd46f27a30%7Cfabd047cff48492a8bbb8f98b9fb9cca%7C0%7C0%7C638744729479743155%7CUnknown%7CTWFpbGZsb3d8eyJFbXB0eU1hcGkiOnRydWUsIlYiOiIwLjAuMDAwMCIsIlAiOiJXaW4zMiIsIkFOIjoiTWFpbCIsIldUIjoyfQ%3D%3D%7C0%7C%7C%7C&sdata=e8ZiQmmZa2zLZH9ok6oqygxTe691MyaBTgj3GIghsJM%3D&reserved=0) | - | - | - | - |
| Caldas | <https://saluddecaldas.gov.co/documentos/>  <https://saluddecaldas.gov.co/documentos/855/boletines/> | - | - | Endemic channel graph presenting: alarm zone, safety zone, success zone and cases. | - |
| Caqueta | [https://www.caqueta.gov.co/covid19/secretaria-de-salud--boletin-epidemiologico-i-2021](https://nam10.safelinks.protection.outlook.com/?url=https%3A%2F%2Fwww.caqueta.gov.co%2Fcovid19%2Fsecretaria-de-salud--boletin-epidemiologico-i-2021&data=05%7C02%7Cmc.tavera%40uniandes.edu.co%7Ce8e2ba40805743388a7508dd46f27a30%7Cfabd047cff48492a8bbb8f98b9fb9cca%7C0%7C0%7C638744729479758669%7CUnknown%7CTWFpbGZsb3d8eyJFbXB0eU1hcGkiOnRydWUsIlYiOiIwLjAuMDAwMCIsIlAiOiJXaW4zMiIsIkFOIjoiTWFpbCIsIldUIjoyfQ%3D%3D%7C0%7C%7C%7C&sdata=UjyVi61NWNMdVHp4dx%2BzP2tWNnFq%2BTYqmf5Yk6DVQQ4%3D&reserved=0)  <https://www.caqueta.gov.co/buscar?q=boletin%20epidemiologico> | Endemic channel graph presenting:  9 - 24 weeks: percentile 25 and 75, median, and cases.  25 - 48 weeks: upper and lower limit confidence interval 95%, geometric mean and cases. | Endemic channel graph presenting:  01 - 52 weeks: upper and lower limit confidence interval 95%, geometric mean and cases. | Endemic channel graph presenting:  01 - 48 weeks: upper and lower limit confidence interval 95%, geometric mean and cases. | - |
| Casanare | <https://www.casanare.gov.co/Dependencias/Salud/Paginas/Boletines-Epidemiologicos.aspx> | 1st - 17th week, endemic channel graph presenting: upper and lower limit confidence interval 95%, stational threshold and cases. Also, other graph indicating with colors: control, expected and alarm.  18th - 31st week, endemic channel graph presenting: upper and lower limit confidence interval 95%, stational threshold and cases.  The endemic channels were constructed with the quartile method and the Bortman method by determining the geometric mean of the historical rates and their confidence interval for dengue. | 1st - 30th week, endemic channel graph presenting: upper and lower limit confidence interval 95%, stational threshold and cases. Also, other graph indicating with colors: control, expected and alarm. | 1st - 30th week, endemic channel graph presenting: upper and lower limit confidence interval 95%, stational threshold and cases.  Also, other graph indicating with colors: control, expected and alarm. | 1st - 30th week, endemic channel graph presenting:  upper and lower limit confidence interval 95%, stational threshold and cases. Also, other graph indicating with colors: control, expected and alarm. |
| Cauca | <https://saludcauca.gov.co/la-secretaria/procesos/misionales/proceso-vigilancia-en-salud-publica/99-boletin-epidemiologico> | - | Endemic channel graph presenting: success, safety and alarm zones; and cases. | Special report: The endemic channel was carried out using Marcelo Bortman's methodology to determine the geometric mean of historical rates and its confidence interval (95% CI), defining control limits as follows: the success zone, below the lower CI; the safety zone, between IC Lower and the middle; the alert zone, between the middle curve, the Upper IC, and the epidemic zone, above the upper IC.  Other reports show the endemic channel graph with: success, safety and alarm zones; and cases. | Endemic channel graph presenting: success, safety and alarm zones; and cases. |
| Cesar | - | - | - | - | - |
| Choco | <https://gestiondelriesgovigilancia.blogspot.com/2022/06/boletines-epidemiologicos-semanale.html> | Endemic channel graph presenting: upper and lower limit, observed ratio and ideal ratio. | - | - | - |
| Cordoba | <https://www.cordoba.gov.co/documentos/2015/boletines-epidemiologicos/> | - | - | - | - |
| Cundinamarca | [https://drive.google.com/drive/folders/1NrTsmYacXj-qgYYb5t_wRfH49Oreip6V](https://nam10.safelinks.protection.outlook.com/?url=https%3A%2F%2Fdrive.google.com%2Fdrive%2Ffolders%2F1NrTsmYacXj-qgYYb5t_wRfH49Oreip6V&data=05%7C02%7Cmc.tavera%40uniandes.edu.co%7Ce8e2ba40805743388a7508dd46f27a30%7Cfabd047cff48492a8bbb8f98b9fb9cca%7C0%7C0%7C638744729479804979%7CUnknown%7CTWFpbGZsb3d8eyJFbXB0eU1hcGkiOnRydWUsIlYiOiIwLjAuMDAwMCIsIlAiOiJXaW4zMiIsIkFOIjoiTWFpbCIsIldUIjoyfQ%3D%3D%7C0%7C%7C%7C&sdata=WfBs%2BwRPHwA2Oe4nx%2FFdlF5zjyXCl4RUXhIFQYsgD9o%3D&reserved=0) |  |  |  |  |
| Guainía | [https://www.guainia.gov.co/tema/secretaria-de-salud/boletines-epidemiologicos](https://nam10.safelinks.protection.outlook.com/?url=https%3A%2F%2Fwww.guainia.gov.co%2Ftema%2Fsecretaria-de-salud%2Fboletines-epidemiologicos&data=05%7C02%7Cmc.tavera%40uniandes.edu.co%7Ce8e2ba40805743388a7508dd46f27a30%7Cfabd047cff48492a8bbb8f98b9fb9cca%7C0%7C0%7C638744729479819775%7CUnknown%7CTWFpbGZsb3d8eyJFbXB0eU1hcGkiOnRydWUsIlYiOiIwLjAuMDAwMCIsIlAiOiJXaW4zMiIsIkFOIjoiTWFpbCIsIldUIjoyfQ%3D%3D%7C0%7C%7C%7C&sdata=sTxgVM8Z0v2ylKsW0yOQAfK2FN0taVoppRUucTNH7YE%3D&reserved=0) | 4th-8th week, endemic channel graph presenting: upper and lower limit, observed ratio and expected ratio. Comparing historic behaviour from 2015 to 2022.  9th-12th week, endemic channel graph presenting: success, safety and alert zones; and cases. Comparing historic behaviour from 2015 to 2022.  13th-16th week, endemic channel graph presenting: upper and lower limit, observed ratio and expected ratio. Comparing historic behaviour from 2014 to 2022.  17th-20th week, endemic channel graph presenting: cases, percentile 25, percentile 75 and mean. Comparing historic behaviour from 2014 to 2022.  21th-44th week, endemic channel graph presenting: upper and lower limit, observed ratio and expected ratio. Comparing historic behaviour from 2017 to 2022. | - | - | - |
| Guaviare | - | - | - | - | - |
| Huila | <https://www.huila.gov.co/documentos/buscar/?q=boletines&id=860> | 1st-48th week,  endemic channel graph presenting: cases, percentile 25, percentile 75 and median. | 1st-48th week,  endemic channel graph presenting: cases, percentile 25, percentile 75 and median. | 44th-52nd week,  endemic channel graph presenting: cases, percentile 25, percentile 50 and percentile 75.  53rd week,  endemic channel graph presenting: upper and lower limit confidence interval 95%, geometric mean and cases. | 1-52nd week,  endemic channel graph presenting: cases, percentile 25, percentile 50 and percentile 75. |
| La Guajira | [http://www.salud-laguajira.gov.co/tema/eventos-de-vigilancia-499539](https://nam10.safelinks.protection.outlook.com/?url=http%3A%2F%2Fwww.salud-laguajira.gov.co%2Ftema%2Feventos-de-vigilancia-499539&data=05%7C02%7Cmc.tavera%40uniandes.edu.co%7Ce8e2ba40805743388a7508dd46f27a30%7Cfabd047cff48492a8bbb8f98b9fb9cca%7C0%7C0%7C638744729479860752%7CUnknown%7CTWFpbGZsb3d8eyJFbXB0eU1hcGkiOnRydWUsIlYiOiIwLjAuMDAwMCIsIlAiOiJXaW4zMiIsIkFOIjoiTWFpbCIsIldUIjoyfQ%3D%3D%7C0%7C%7C%7C&sdata=UjrP4xiZZ%2Btg5CZp3yKC1ChjcRxjs3fFBbLbYadRbmA%3D&reserved=0) | 18th week,  endemic channel graph presenting: cases, percentile 25, percentile 75 and median.  19th-22nd week and 48th week,  endemic channel graph presenting: cases, percentile 25 (below expected), percentile 75 (above expected) and median (alert). | - | - | - |
| Magdalena | - | - | - | - | - |
| Meta | <https://meta.gov.co/documentacion/1421> | 36th week, endemic channel graph presenting: cases, and success, safety and alarm zones. | - | - | - |
| Nariño | [http://idsn.gov.co/site/web2/index.php/subdireccion-de-salud-publica/epidemiologia/429-eventos-de-notificacion-enos](https://nam10.safelinks.protection.outlook.com/?url=http%3A%2F%2Fidsn.gov.co%2Fsite%2Fweb2%2Findex.php%2Fsubdireccion-de-salud-publica%2Fepidemiologia%2F429-eventos-de-notificacion-enos&data=05%7C02%7Cmc.tavera%40uniandes.edu.co%7Ce8e2ba40805743388a7508dd46f27a30%7Cfabd047cff48492a8bbb8f98b9fb9cca%7C0%7C0%7C638744729479893809%7CUnknown%7CTWFpbGZsb3d8eyJFbXB0eU1hcGkiOnRydWUsIlYiOiIwLjAuMDAwMCIsIlAiOiJXaW4zMiIsIkFOIjoiTWFpbCIsIldUIjoyfQ%3D%3D%7C0%7C%7C%7C&sdata=pYJkhLkTtPLNlmNZMaW5Qbvx1oBfrssYCaR6bkizzhg%3D&reserved=0) | - | - | - | - |
| Norte de Santander | - | - | - | - | - |
| Putumayo | [https://www.saludputumayo.gov.co/index.php/saludpublica/epidemiologia](https://nam10.safelinks.protection.outlook.com/?url=https%3A%2F%2Fwww.saludputumayo.gov.co%2Findex.php%2Fsaludpublica%2Fepidemiologia&data=05%7C02%7Cmc.tavera%40uniandes.edu.co%7Ce8e2ba40805743388a7508dd46f27a30%7Cfabd047cff48492a8bbb8f98b9fb9cca%7C0%7C0%7C638744729479908491%7CUnknown%7CTWFpbGZsb3d8eyJFbXB0eU1hcGkiOnRydWUsIlYiOiIwLjAuMDAwMCIsIlAiOiJXaW4zMiIsIkFOIjoiTWFpbCIsIldUIjoyfQ%3D%3D%7C0%7C%7C%7C&sdata=Uq59ejDL3hdJk3UyzMIZsNKyZw1JJWaoDJhFRTmYZqw%3D&reserved=0) | - | 1st-52nd week, endemic channel graph presenting: upper and lower limit confidence interval, geometric mean and cases. Comparing historic behaviour from 2014 to 2020. | 1st-53rd week, endemic channel graph presenting: upper and lower limit confidence interval, geometric mean and cases. Comparing historic behaviour:  1st-4th week: 2011-2019.  8th-52th week: 2013 to 2019. | - |
| Quindio | [https://quindio.gov.co/salud-publica/salud-publica-3](https://nam10.safelinks.protection.outlook.com/?url=https%3A%2F%2Fquindio.gov.co%2Fsalud-publica%2Fsalud-publica-3&data=05%7C02%7Cmc.tavera%40uniandes.edu.co%7Ce8e2ba40805743388a7508dd46f27a30%7Cfabd047cff48492a8bbb8f98b9fb9cca%7C0%7C0%7C638744729479922606%7CUnknown%7CTWFpbGZsb3d8eyJFbXB0eU1hcGkiOnRydWUsIlYiOiIwLjAuMDAwMCIsIlAiOiJXaW4zMiIsIkFOIjoiTWFpbCIsIldUIjoyfQ%3D%3D%7C0%7C%7C%7C&sdata=Dl8VDWhBC2oOrlLCo3mmSmEbGd7sis5fr4MaV8Glorg%3D&reserved=0) | - | - | - | - |
| Risaralda | <https://www.risaralda.gov.co/salud/documentos/150511/boletines-epidemiologicos/?genPag=1> | - | - | - | - |
| Santander | [https://santander.gov.co/publicaciones/7535/boletines-epidemiologicos/](https://nam10.safelinks.protection.outlook.com/?url=https%3A%2F%2Fsantander.gov.co%2Fpublicaciones%2F7535%2Fboletines-epidemiologicos%2F&data=05%7C02%7Cmc.tavera%40uniandes.edu.co%7Ce8e2ba40805743388a7508dd46f27a30%7Cfabd047cff48492a8bbb8f98b9fb9cca%7C0%7C0%7C638744729479949406%7CUnknown%7CTWFpbGZsb3d8eyJFbXB0eU1hcGkiOnRydWUsIlYiOiIwLjAuMDAwMCIsIlAiOiJXaW4zMiIsIkFOIjoiTWFpbCIsIldUIjoyfQ%3D%3D%7C0%7C%7C%7C&sdata=dpKr%2FRvqYr%2FIM1hfCj2cflkVWXbGQvkJjMunyEAIHM0%3D&reserved=0) | 6th-42nd week, endemic channel graph presenting: cases, and success, safety and alert zones. | - | - | 24th-49th. week, endemic channel graph presenting: cases, and success, safety and alarm zones. |
| Sucre | <https://www.saludsucre.gov.co/tema/boletines-epidemiologicos> | 9th-12th week,  endemic channel graph presenting: cases, percentile 25, percentile 50 and percentile 75 | - | - | - |
| Tolima | [http://www.saludtolima.gov.co/bsivigila/](https://nam10.safelinks.protection.outlook.com/?url=http%3A%2F%2Fwww.saludtolima.gov.co%2Fbsivigila%2F&data=05%7C02%7Cmc.tavera%40uniandes.edu.co%7Ce8e2ba40805743388a7508dd46f27a30%7Cfabd047cff48492a8bbb8f98b9fb9cca%7C0%7C0%7C638744729479976743%7CUnknown%7CTWFpbGZsb3d8eyJFbXB0eU1hcGkiOnRydWUsIlYiOiIwLjAuMDAwMCIsIlAiOiJXaW4zMiIsIkFOIjoiTWFpbCIsIldUIjoyfQ%3D%3D%7C0%7C%7C%7C&sdata=4TZbU8RBesQ4TgqzfRVH5K04VfLQxy%2B%2F9%2FTrGxHS8iw%3D&reserved=0) | - | - | - | - |
| Valle del Cauca | [https://www.valledelcauca.gov.co/salud/publicaciones/73894/boletin-epidemiologico-ano-2022/](https://nam10.safelinks.protection.outlook.com/?url=https%3A%2F%2Fwww.valledelcauca.gov.co%2Fsalud%2Fpublicaciones%2F73894%2Fboletin-epidemiologico-ano-2022%2F&data=05%7C02%7Cmc.tavera%40uniandes.edu.co%7Ce8e2ba40805743388a7508dd46f27a30%7Cfabd047cff48492a8bbb8f98b9fb9cca%7C0%7C0%7C638744729479990283%7CUnknown%7CTWFpbGZsb3d8eyJFbXB0eU1hcGkiOnRydWUsIlYiOiIwLjAuMDAwMCIsIlAiOiJXaW4zMiIsIkFOIjoiTWFpbCIsIldUIjoyfQ%3D%3D%7C0%7C%7C%7C&sdata=PBHciokPtdsFNoWi9H7Fj7Lt0IdGzuuqKHFHpvpgmVM%3D&reserved=0) | 1st-12th week, endemic channel with geometric mean and historical data from 2014-2021.  13th-52th week, endemic channel with mean and historical data from 2014-2021. | Endemic channel with geometric mean and historical from 2013 - 2020 | 1st - 3rd: No endemic channel  4th-49th: Endemic channel with geometric mean with historical from 2013-2019.  50th-53th: Endemic channel with geometric mean with historical from 2013-2017. | 1rst-4th week, endemic channel with geometric mean with historical from 2012-2018.  5th-43th endemic channel with expected and observed ratio and historical from 2013-2018.  42th don´t specify the method (razón ideal) with historical from 2012-2018.  45th-52th week, endemic channel with geometric mean with historical from 2012-2018. |
| Vaupes | [https://www.vaupes.gov.co/tema/salud-publica](https://nam10.safelinks.protection.outlook.com/?url=https%3A%2F%2Fwww.vaupes.gov.co%2Ftema%2Fsalud-publica&data=05%7C02%7Cmc.tavera%40uniandes.edu.co%7Ce8e2ba40805743388a7508dd46f27a30%7Cfabd047cff48492a8bbb8f98b9fb9cca%7C0%7C0%7C638744729480003780%7CUnknown%7CTWFpbGZsb3d8eyJFbXB0eU1hcGkiOnRydWUsIlYiOiIwLjAuMDAwMCIsIlAiOiJXaW4zMiIsIkFOIjoiTWFpbCIsIldUIjoyfQ%3D%3D%7C0%7C%7C%7C&sdata=cbhvffPW4MSdhLUyKyuFXwjEBmzonWwwEFGmX%2F7PXoM%3D&reserved=0) | - | - | - | - |
| Vichada | <http://www.vichada.gov.co/indicadores/boletines-epidemiologicos-de-salud-ano-2018> | - | - | - | - |

Assessment of the retrospective window

Supplementary Table 5. LME model for the retrospective windows experiment. Percentage of change of the epidemiological warning threshold displayed by the endemic channels when different lengths of retrospective data were used. The ▴ symbol refers to not significant treatments (p-value > 0.01).

| Transmission level | Experiment (Window) | Intercept | Effect | Epidemiological Threshold |
| --- | --- | --- | --- | --- |
| Low | 5 | 0,12 | -0,004 | -3,58% |
|  | 6▴ | 0,12 | 0,000 | -0,27% |
|  | 8 | 0,12 | -0,001 | -1,18% |
|  | 9 | 0,12 | -0,004 | -3,46% |
| Medium | 5 | 0,54 | -0,010 | -1,88% |
|  | 6▴ | 0,54 | -0,001 | -0,18% |
|  | 8 | 0,54 | -0,010 | -1,79% |
|  | 9 | 0,54 | -0,028 | -5,21% |
| High | 5 | 1,64 | -0,040 | -2,42% |
|  | 6 | 1,64 | -0,009 | -0,56% |
|  | 8 | 1,64 | -0,032 | -1,97% |
|  | 9 | 1,64 | -0,088 | -5,39% |
| Very-high | 5 | 10,68 | -0,460 | -4,31% |
|  | 6 | 10,68 | -0,186 | -1,74% |
|  | 8 | 10,68 | -0,108 | -1,01% |
|  | 9 | 10,68 | -0,425 | -3,98% |

Supplementary Table 6. Endemic channel performance for the retrospective windows experiment (p-value < 0.01 *, p-value < 0.001 **, p-value < 0.0001 ***).

| Transmission level | Experiment (Window) | Outbreak detection capacity | Effective response rate | Endemic channel performance |
| --- | --- | --- | --- | --- |
| Low | *** Window 5 | 0,04 | 0,26 | 0,19 |
|  | * Window 6 | 0,03 | 0,28 | 0,20 |
|  | Window 7 | 0,02 | 0,28 | 0,19 |
|  | * Window 8 | 0,01 | 0,26 | 0,17 |
|  | ** Window 9 | 0,01 | 0,26 | 0,17 |
| Medium | ** Window 5 | 0,23 | 0,29 | 0,27 |
|  | ** Window 6 | 0,23 | 0,30 | 0,27 |
|  | Window 7 | 0,20 | 0,26 | 0,24 |
|  | * Window 8 | 0,17 | 0,28 | 0,24 |
|  | *** Window 9 | 0,15 | 0,28 | 0,23 |
| High | ** Window 5 | 0,57 | 0,28 | 0,43 |
|  | Window 6 | 0,54 | 0,27 | 0,41 |
|  | Window 7 | 0,52 | 0,27 | 0,40 |
|  | Window 8 | 0,50 | 0,28 | 0,39 |
|  | Window 9 | 0,48 | 0,30 | 0,39 |
| Very-high | Window 5 | 0,88 | 0,29 | 0,60 |
|  | Window 6 | 0,88 | 0,28 | 0,59 |
|  | Window 7 | 0,84 | 0,30 | 0,59 |
|  | * Window 8 | 0,77 | 0,29 | 0,55 |
|  | *** Window 9 | 0,75 | 0,30 | 0,53 |

Assessment of the previous outbreaks handling

Supplementary Table 7. LME model for the previous outbreaks handling experiment. Percentage of change of the epidemiological warning threshold displayed by the endemic channels when different strategies were used. All results were statistically significant (p-value < 0.01).

| Transmission level | Experiment  (Previous outbreaks handling) | Intercept | Effect | Epidemiological Threshold |
| --- | --- | --- | --- | --- |
| Low | Including outliers | 0,12 | 0,002 | 1,63% |
|  | Replacing outliers | 0,12 | 0,000 | 0,17% |
| Medium | Including outliers | 0,52 | 0,025 | 4,74% |
|  | Replacing outliers | 0,52 | 0,003 | 0,57% |
| High | Including outliers | 2 | 0,054 | 3,40% |
|  | Replacing outliers | 2 | 0,006 | 0,40% |
| Very-high | Including outliers | 10,53 | 0,118 | 1,12% |
|  | Replacing outliers | 10,53 | 0,010 | 0,10% |

Supplementary Table 8. Endemic channel performance for the previous outbreaks handling experiment (p-value < 0.01 *, p-value < 0.001 **, p-value < 0.0001 ***).

| Transmission level | Experiment  (Previous outbreaks handling) | Outbreak detection capacity | Effective response rate | Endemic channel performance |
| --- | --- | --- | --- | --- |
| Low | Ignore outliers | 0,08 | 0,25 | 0,17 |
|  | * Including outliers | 0,07 | 0,22 | 0,14 |
|  | * Replacing outliers | 0,07 | 0,24 | 0,16 |
| Medium | Ignore outliers | 0,20 | 0,28 | 0,24 |
|  | * Including outliers | 0,20 | 0,29 | 0,24 |
|  | *** Replacing outliers | 0,19 | 0,30 | 0,24 |
| High | Ignore outliers | 0,57 | 0,34 | 0,46 |
|  | Including outliers | 0,58 | 0,35 | 0,47 |
|  | * Replacing outliers | 0,56 | 0,35 | 0,46 |
| Very-high | Ignore outliers | 0,82 | 0,36 | 0,59 |
|  | Including outliers | 0,81 | 0,36 | 0,58 |
|  | Replacing outliers | 0,81 | 0,38 | 0,59 |

Effect of zero-values handling

Supplementary Table 9. LME model for the zero-values handling experiment. Percentage of change of the epidemiological warning threshold displayed by the endemic channels when different strategies were used. All results were statistically significant (p-value < 0.01).

| Transmission level | Experiment  (Zero values handling) | Intercept | Effect | Epidemiological Threshold |
| --- | --- | --- | --- | --- |
| Low | Ignoring values | 0,12 | 1,521 | 1295,57% |
|  | Minimal shift | 0,12 | -0,012 | -10,00% |
|  | Weighting values | 0,12 | 0,161 | 137,48% |
| Medium | Ignoring values | 0,52 | 1,527 | 294,28% |
|  | Minimal shift | 0,52 | -0,324 | -62,44% |
|  | Weighting values | 0,52 | 0,215 | 41,49% |
| High | Ignoring values | 2 | 1,284 | 81,21% |
|  | Minimal shift | 2 | -0,858 | -54,28% |
|  | Weighting values | 2 | 0,294 | 18,58% |
| Very-high | Ignoring values | 10,53 | 0,607 | 5,76% |
|  | Minimal shift | 10,53 | -0,977 | -9,28% |
|  | Weighting values | 10,53 | 0,077 | 0,73% |

Supplementary Table 10. Endemic channel performance for the zero-values handling experiment (p-value < 0.01 *, p-value < 0.001 **, p-value < 0.0001 ***).

| Transmission level | Experiment  (Zero values handling) | Outbreak detection capacity | Effective response rate | Endemic channel performance |
| --- | --- | --- | --- | --- |
| Low | Shift values | 0,08 | 0,25 | 0,17 |
|  | Ignoring values | 0,11 | 0,20 | 0,15 |
|  | *** Minimal shift | 0,26 | 0,10 | 0,18 |
|  | *** Weighting values | 0,04 | 0,23 | 0,14 |
| Medium | Shift values | 0,20 | 0,28 | 0,24 |
|  | Ignoring values | 0,23 | 0,28 | 0,26 |
|  | *** Minimal shift | 0,59 | 0,14 | 0,36 |
|  | *** Weighting values | 0,12 | 0,29 | 0,20 |
| High | Shift values | 0,57 | 0,34 | 0,46 |
|  | Ignoring values | 0,55 | 0,29 | 0,42 |
|  | *** Minimal shift | 0,89 | 0,13 | 0,51 |
|  | *** Weighting values | 0,39 | 0,34 | 0,36 |
| Very-high | Shift values | 0,82 | 0,36 | 0,59 |
|  | Ignoring values | 0,83 | 0,33 | 0,58 |
|  | *** Minimal shift | 0,98 | 0,29 | 0,64 |
|  | * Weighting values | 0,78 | 0,36 | 0,57 |

The effect of administrative aggregation in the endemic channel performance

Supplementary Table 11. Departmental endemic channel performance for the retrospective windows experiment. No treatment had a significant effect (p-value < 0.01).

| Transmission level | Experiment (Window) | Outbreak detection capacity | Effective response rate | Endemic channel performance |
| --- | --- | --- | --- | --- |
| Low | Window 5 | 0,94 | 0,54 | 0,74 |
|  | Window 6 | 0,91 | 0,55 | 0,73 |
|  | Window 7 | 0,90 | 0,49 | 0,70 |
|  | Window 8 | 0,79 | 0,44 | 0,62 |
|  | Window 9 | 0,86 | 0,52 | 0,69 |
| Medium | Window 5 | 0,79 | 0,43 | 0,61 |
|  | Window 6 | 0,78 | 0,44 | 0,61 |
|  | Window 7 | 0,71 | 0,45 | 0,58 |
|  | Window 8 | 0,69 | 0,45 | 0,57 |
|  | Window 9 | 0,75 | 0,52 | 0,63 |
| High | Window 5 | 0,86 | 0,31 | 0,58 |
|  | Window 6 | 0,88 | 0,33 | 0,60 |
|  | Window 7 | 1,00 | 0,42 | 0,71 |
|  | Window 8 | 0,77 | 0,38 | 0,58 |
|  | Window 9 | 0,90 | 0,35 | 0,63 |
| Very-high | Window 5 | 1,00 | 0,43 | 0,71 |
|  | Window 6 | 0,98 | 0,39 | 0,69 |
|  | Window 7 | 1,00 | 0,35 | 0,68 |
|  | Window 8 | 1,00 | 0,36 | 0,68 |
|  | Window 9 | 0,99 | 0,43 | 0,71 |

Supplementary Table 12. Departmental endemic channel performance for the previous outbreaks handling experiment. No treatment had a significant effect (p-value < 0.01).

| Transmission level | Experiment  (Previous outbreaks handling) | Outbreak detection capacity | Effective response rate | Endemic channel performance |
| --- | --- | --- | --- | --- |
| Low | Ignore outliers | 0,93 | 0,43 | 0,68 |
|  | Including outliers | 0,92 | 0,47 | 0,70 |
|  | Replacing outliers | 0,92 | 0,48 | 0,70 |
| Medium | Ignore outliers | 0,69 | 0,44 | 0,56 |
|  | Including outliers | 0,69 | 0,41 | 0,55 |
|  | Replacing outliers | 0,69 | 0,46 | 0,57 |
| High | Ignore outliers | 0,93 | 0,38 | 0,65 |
|  | Including outliers | 0,88 | 0,41 | 0,64 |
|  | Replacing outliers | 0,93 | 0,39 | 0,66 |
| Very-high | Ignore outliers | 0,96 | 0,42 | 0,69 |
|  | Including outliers | 0,94 | 0,42 | 0,68 |
|  | Replacing outliers | 0,96 | 0,43 | 0,69 |

Supplementary Table 13. Departmental endemic channel performance for the zero-values handling experiment. No treatment had a significant effect (p-value < 0.01).

| Transmission level | Experiment  (Zero values handling) | Outbreak detection capacity | Effective response rate | Endemic channel performance |
| --- | --- | --- | --- | --- |
| Low | Shift values | 0,93 | 0,43 | 0,68 |
|  | Ignoring values | 0,82 | 0,32 | 0,57 |
|  | Minimal shift | 0,92 | 0,30 | 0,61 |
|  | Weighting values | 0,88 | 0,48 | 0,68 |
| Medium | Shift values | 0,69 | 0,44 | 0,56 |
|  | Ignoring values | 0,72 | 0,42 | 0,57 |
|  | Minimal shift | 0,92 | 0,37 | 0,64 |
|  | Weighting values | 0,69 | 0,43 | 0,56 |
| High | Shift values | 0,93 | 0,38 | 0,65 |
|  | Ignoring values | 0,95 | 0,49 | 0,72 |
|  | Minimal shift | 1,00 | 0,32 | 0,63 |
|  | Weighting values | 0,73 | 0,44 | 0,59 |
| Very-high | Shift values | 0,96 | 0,42 | 0,69 |
|  | Ignoring values | 0,96 | 0,42 | 0,69 |
|  | Minimal shift | 0,99 | 0,43 | 0,71 |
|  | Weighting values | 0,98 | 0,43 | 0,70 |
